# Evaluating the impact of health awareness events on Google search frequency

**DOI:** 10.1016/j.pmedr.2019.100887

**Published:** 2019-05-20

**Authors:** Zheng Hao, Miao Liu, Xijin Ge

**Affiliations:** a State University of New York at Oswego, United States of America; b South Dakota State University, United States of America

**Keywords:** Google Trend, Health awareness events, Transfer function noise modeling

## Abstract

Over two hundred health awareness events take place in the United States in order to educate the public about various diseases. It would be informative and instructive for the organizations to know the impact of these events, although such information could be difficult to measure. We investigated whether 46 selected events attract the public attention by increasing the search frequencies of certain keywords. Internet search data from 2004 to 2017 were downloaded from Google Trend (GT). Three statistical methods including Transfer Function Noise modeling, Wilcoxon Rank Sum test, and Binomial inference were conducted. Our study showed that 10 health awareness events resulted in increased search frequencies in the event months, and 28 events did not, with the rest being classified as unclear.

## 1 Introduction

### 1.1 Background

Chronic diseases cause 70*%* of deaths in the United States every year, even though many of those diseases are preventable (CDC, 2014). The goal of holding health awareness events is to raise attention and educate the public about diseases. Take the National Breast Cancer awareness month as an example: the National Breast Cancer Foundation devotes efforts to educating women on early detection to reduce the risk of breast cancer, helping those diagnosed with breast cancer, as well as raising funds to support research. Companies join the National Breast Cancer Awareness Month to help improve awareness of breast cancer and raise funds for medical research (Centers for Disease Control and Prevention, 2008). Wang and et al. (2018) conducted statistically analysis and tests on the relationship between health education and behaviors toward infectious diseases in different countries. One of their results was that populations exposed to different health education had significantly different preventive behavior toward infectious diseases. Neuner-Jehle et al. (2013) found that well-structured program of counselling could increase patients' favorable health behaviors.

It is estimated that 97*%* of the information flowing through two-way telecommunication were carried by the Internet by 2007 (Hilbert and Lopez, 2011). The number of Internet users has increased enormously and surpasses 3 billion or about 46.1*%* of the world population in 2014 (Internet Society (2014)). Google has led the U.S. core search market for the past decade (comScore), and millions of people worldwide use it to search for health topics every day (Johnson et al., 2004, Carneiro and Mylonakis, 2009). In particular it occupied three quarters of the search engine market in 2017.

Our objective was to determine if health awareness events resulted in higher Google search frequencies, which could be evidence for increased public awareness. The results could benefit a variety of parties, for instance, the Department of Public Health and public interest groups could optimally rearrange resources allocation among events.

### 1.2 Related work

Using Internet statistics to explain and predict quantities has been popular among researcher. Bollen et al. (2011) classified tweets into different moods to quantify the daily public mood and used it to predict stock market by using different models. The idea was based on the fact that people intentionally or unintentionally disclosed their thinking online by some means including social media such as Twitter, which might be a factor of stock price variation. What was interesting was that the authors used tweets which was not traditionally considered as an economic factor unlike some classical factors such as interest rates, GDP, and unemployment rates.

Ginsberg et al. (2009), Doornik (2009) and Carneiro and Mylonakis proved that Google Trends data could be predictive for current influenza-like activity levels by 1–2 weeks earlier before conventional centers for disease control and prevention surveillance systems by comparing GT data and the actual disease numbers and provided different case studies. The search frequency would dramatically increase before and during the disease outbreak. Similarly, Cook et al. (2011) chose H1N1 ease cases. The increasing search frequency could be useful in identifying the presence of diseases and the media effect on web users' search behaviors (Eysenbach, 2006).

GT data was proven to be effective in terms of modeling other areas such as marketing and information security. Youn and Cho (2016) used GT data and Autoregressive Integrated Moving Average (ARIMA) models to conduct nowcast for TV market of a few brands and was able reveal the correlation. Accurate prediction for the near future of the market was obtained. Rech (2007i) used GT data to analyze the attention that products received and the cause-effect relation among a few factors in software engineering. Mondal and Wasimi (2005) used transfer function noise model to study the effect of monthly rain fall on the Ganges River flow, with both data sets being time series. In our case, we will use an impulse series as the explanatory.

Shariatpanahi et al. (2017) used GT data to assess the impact of disease awareness program by dynamics modeling the GT data which considers two ways of being aware of diseases, one of which was people's communication and the other was by health events. They studied four diseases with corresponding events and quantitatively estimated the strength of the impact of the events. Their interest was on the daily worldwide events instead of monthly events.

Seifter et al. (2010) show that GT data was high related to the public attention on diseases according to a study on Lyme disease. Jacobsen and Jacobsen (2011) analyzed the number of articles published and number of early detection of disease in the event month for breast cancer and concluded that the event did promote public attention. The study quantitatively indicated that a successful event actually educated public and encouraged early detection. In Ayers and Althouse (2016), Ayers et al. studied the Great American Smokeout health awareness event by using a number of data sets such as number of news, tweets, Wiki visits and etc. Their proposed evaluation method for event effectiveness was to first fit counterfactual data by assuming the event had not occurred, then compare them with the actual data. Although their approach was quantitative, they used the percent change where it is unclear detect the threshold of significance.

## 2 Datasets and preprocessing

### 2.1 Datasets

A set of 46 monthly events were selected from the event list on the website of healthline (Healthline awareness directory) as of 2017. We only focused on monthly events which were closely related to disease, since we would like to match the time interval for GT data. Since GT data was based on the search frequency of one or a few words which we called a query, we selected a query for each event and presented them in Appendix A. In fact, for some events, there were more than one meaningful queries, then we picked the one with highest frequency.

**Fig. 1:**
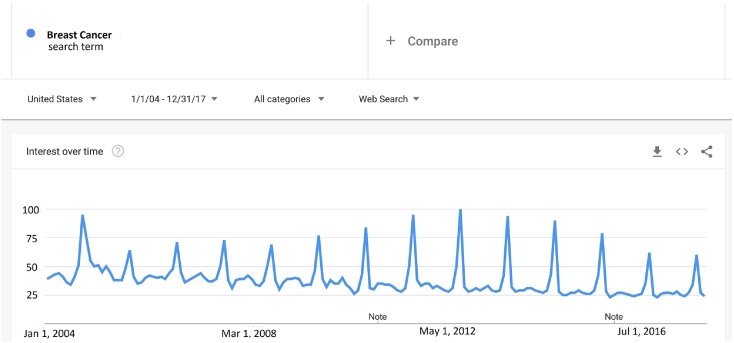
Google Trends search plot for the query of breast cancer.

On Google Trends webpage, users are able to track the search popularity of queries in different languages across regions starting from January 2004. Weekly or monthly GT data may be downloaded as a CSV file depending on the total time range. Since the pure values of queries can be huge numbers, Google rescales them in a range from 0 to 100 with the highest frequency being 100. Four options, including Region, Time, Category and Search Type are needed to specify a search and are selected as United State, 2004–2017, Health, and Web search respectively in this work. Fig. 1 showed the query of Breast Cancer as a time series plot.

### 2.2 Data preprocessing

Monthly data from 2004 to 2017 for 46 selected queries were collected. All data points were integers between 0 and 100, with no missing data. We rescaled every month to an equal length of 30 days to reduce the variation caused by uneven number of days. Specifically, January, March, May, July, August, October, and December data points were multiplied by $\frac{30}{31}$, and February data points were multiplied by $\frac{30}{28}$.

## 3 Methodology

In this chapter, we proposed three different quantitative methods to evaluate the impact as well as their thresholds. The main method was to use transfer function noise modeling with impulse series as input. Then inferences based on Wilcoxon Rank Sum test and Binomial distribution were used to consolidate results.

### 3.1 Transfer function noise model

The (Seasonal) Autoregressive Integrated Moving Average models (ARIMA or SARIMA) make interpretation and forecast by developing the intrinsic pattern of the single response time series. The Google Search data forms a time series. If it behaves completely with its intrinsic pattern, it should be modeled by an ARIMA or SARIMA process. All events are month-long which are just equal to one period of the time series data, therefore they could be viewed as an impulse series with impulse taking place once every 12 periods. Then the idea becomes to test if the impulse series has significant effect on the original series.

A general SARIMA (*p*,*d*,*q*)(*P*,*D*,*Q*)_*s*_ has the form:

$$\begin{array}{c} \left(1-\overset{P}{\underset{i=1}{\sum }}\phi_{i}^{*}B^{is}\right)\left(1-\overset{p}{\underset{i=1}{\sum }}\phi_{i}B^{i}\right){\left(1-B^{s}\right)}^{D}{\left(1-B\right)}^{d}y_{t}\\ =\left(1+\overset{Q}{\underset{i=1}{\sum }}\theta_{i}^{*}B^{is}\right)\left(1+\overset{q}{\underset{i-1}{\sum }}\theta_{i}B^{i}\right)\epsilon_{t}, \end{array}$$

where *B* is the backshift operator, *By*_*t*_ = *y*_*t*−1_, *ϵ*_*t*_ is a white noise, and *ϕ*_*i*_, *θ*_*i*_, $\phi_{i}^{*}$, and $\theta_{i}^{*}$ are constant coefficients. This model can be expressed by a more compact notation as:

$$\phi (B)y_{t}=\theta (B)\epsilon_{t}\;⟷\;y_{t}=\frac{\theta (B)}{\phi (B)}\epsilon_{t}$$

If there is another series, say {*x*_*t*_} which is called an input series that has a relationship with {*y*_*t*_}. The Transfer Function Noise Model is built to describe this situation as

(3.1) $$y_{t}=c+\frac{w(B)B^{b}}{\delta (B)}x_{t}+\frac{\theta (B)}{\phi (B)}\epsilon_{t}$$

Let {*x*_*t*_} be an impulse time series with *x*_*i*_ = 0 if it corresponds a non event month, and *x*_*i*_ = 1 if it corresponds an event month. Eq. (3.1) is called the Intervention model, whose operator $\frac{w(B)B^{b}}{\delta (B)}$ usually has a fairly simple form. Letting $\frac{w(B)B^{b}}{\delta (B)}=w_{0}$ yields

(3.2) $$y_{t}=c+w_{0}x_{t}+\frac{\theta (B)}{\phi (B)}\epsilon_{t}$$

from which one is interested in how much the impulse {*x*_*t*_} contributes to the current response {*y*_*t*_}.

We would first determine whether there was a seasonality in each data set, then fit the best ARIMA/SARIMA model.

Secondly, we would fit a transfer function noise model and use the better one of the following two attempt to determine the orders of *θ*(*B*) and *ϕ*(*B*) in Eq. (3.2).

The first attempt was to use the same order as the ARIMA/SARIMA. In second attempt, we first replaced the event month data with the average of the previous and next month. The idea was that after this replacement, the new data was our best guess for what the data would be if there were no event happening. We used the new data to determine the orders of the ARIMA/SARIMA model and use them in Eq. (3.2). The better attempt was chosen as the final transfer function noise model.

We would conclude that the event contributes to the number of search if the transfer function noise model was better fitted than the ARIMA/SARIMA model, and the parameter *w*_0_ was significant at 0.05 level.

### 3.2 Wilcoxon rank sum test

The Wilcoxon Rank Sum test was introduced by Frank Wilcoxon in Wilcoxon (1945) to compare the means of two groups. Blair and Higgins (1980) showed that Wilson test usually held large power advantages over t test and was asymptotically more efficient than t test. In our case, the sample sizes were unequal and the sample distributions were unclear, thus we believed the Wilcoxon Rank-Sum was more appropriate than the t-test.

Data points were splitted into two groups as event month and non event month, and we set the null hypothesis as the two group of observations coming from the same population. The Wilcoxon test is based upon ranking data points of the combined sample. Assign numeric ranks to all the observations with 1 being the smallest value. If there is a group that ties, assign the rank equal to its average ranking. The Wilcoxon rank-sum test statistic is the sum of the ranks for observations from one of the samples and therefore are calculated as:

(3.3) $$U_{x}=n_{x}n_{y}+\frac{n_{x}(n_{x}+1)}{2}-u_{x}$$

(3.4) $$U_{y}=n_{x}n_{y}+\frac{n_{y}(n_{y}+1)}{2}-u_{y}$$

where *n*_*x*_ and *n*_*y*_ are the two sample sizes; *u*_*x*_ and *u*_*y*_ are the sums of the ranks in samples *x* and *y* respectively. The smaller value between *U*_*x*_ and *U*_*y*_ is the one used to consult significance tables to estimate the p-value.

### 3.3 Inference by binomial distribution

Suppose for a disease, its event brings significantly more attention to the public, we would anticipate the frequencies for the event month to be highest. Therefore, we used the null hypothesis that the search frequencies were completely random. Under the null hypothesis, every month has equal probability 1/12 to be the peak since all selected diseases are not seasonal as an influenza-like illness. Let *k* be the number of yearly peaks for event-month data in 14 years. Among 14 years, the probability that a certain month appears to be the peak *k* times is

P(X=k)=14k112k1−112(14−k),whereX∼B14,112

In particular, *k* = 4 is the largest value making the probability less than 0.05, and *P*(*X* = 4) = 0.02. Therefore, that the event month appears to be the peak at least 4 times indicates evidence that the event-month data is significantly different from the other months.

From a statistical perspective, health awareness events that show evidence of significance in all three method are defined as impactful health awareness events. Health awareness events that have insignificant results for all three tests are defined as unimpactful health awareness events. The events with inconsistent results by different methods are defined as unclear. This study is focused on the information carried by Google Trend data. Of course, having statistical significance results does not necessarily imply that people are taking actions or change their behaviors in a positive direction in practice. Some discussion about limitation is provided in chapter 5.

## 4 Results

Details for two selected events as case study were presented in this chapter. All 46 selected query data were analyzed and ten were concluded to be impactful in raising search frequencies of related diseases including Alcohol Awareness, Autism, Breast Cancer, Colon Cancer, Dental Health, Heart Disease, Immunization, National Nutrition, Ovarian Cancer, and Sids. Eight events were unclear due to inconsistent results and the others were unimpactful. See Table 1 for complete results.

**Table 1:** Results of three methods for all 46 queries. Asterisk means p value < 0.05.

Event	Wilcoxon sum test p-value	Peaks at event months	Transfer function noise model fits better	Input series coefficient p value	Conclusion
Alcohol Awareness	0.0013*	6	Yes	0*	Impactful
Autism	0*	12	Yes	0*	Impactful
Breast Cancer	0*	14	Yes	0*	Impactful
Coloncancer	0.0008*	7	Yes	0.0129*	Impactful
Dental Health	0*	14	Yes	0*	Impactful
Heart Disease	0*	14	Yes	0.0016*	Impactful
Immunization	0*	14	Yes	0.0009*	Impactful
National Nutrition	0*	5	Yes	0.0054*	Impactful
Ovarian Cancer	0.0007*	7	Yes	0*	Impactful
Sids	0.0008*	4	Yes	0*	Impactful
Asthma Allergy	0.0183*	3	Yes	0.0636	Unclear
Diabetes	0.0297*	1	No	0.0813	Unclear
Endometriosis	0.1314	4	No	0.7099	Unclear
Epilepsy	0.0159*	0	No	0.2426	Unclear
Lung Cancer	0.0341*	1	No	0.1929	Unclear
Lupus	0.0192*	4	Yes	0.7506	Unclear
Menopause	0.0177*	2	No	0.5078	Unclear
Skin Cancer	0	5	No	0.0504	Unclear
Alcohol Drug Addiction	0.3959	0	Yes	0.0718	Unimpactful
Alzheimer	0.177	1	No	0.2090	Unimpactful
Amblyopia	0.8139	1	No	0.9164	Unimpactful
Aphasia	0.9809	0	No	0.0009*	Unimpactful
Arthritis	0.1718	1	No	0.6986	Unimpactful
Birth Defect	0.1899	0	No	0.5783	Unimpactful
Celiac	0.22	1	No	0.7075	Unimpactful
Cervical	0.8439	0	No	0.0012*	Unimpactful
Cholesterol	0.2667	1	No	0.0124*	Unimpactful
Dental Hygiene	0.0724	1	No	0.5741	Unimpactful
Depression	0.1168	1	No	0*	Unimpactful
Down Syndrome	0.2446	1	No	0.0484*	Unimpactful
Eye Injury	0.4793	0	Yes	0.2093	Unimpactful
Glaucoma	0.6872	0	Yes	0.0274*	Unimpactful
Hepatitis	0.3914	0	Yes	0.0300*	Unimpactful
High Blood Pressure	0.8289	0	No	0.0038*	Unimpactful
Ibs	0.1389	1	No	0.0033*	Unimpactful
Leukemia	0.249	0	No	0.0024*	Unimpactful
Mental Health	0.5126	0	No	0*	Unimpactful
Osteoporosis	0.6779	0	No	0.0429*	Unimpactful
Pancreatic Cancer	0.2508	0	Yes	0.6771	Unimpactful
Prostate	0.7092	0	No	0.6659	Unimpactful
Psoriasis	0.8311	0	No	0.3862	Unimpactful
Sclerosis	0.1822	0	No	0.0258*	Unimpactful
Scoliosis	0.3892	1	Yes	0.4533	Unimpactful
Spina Bifida	0.0036*	2	No	0.0047*	Unimpactful
Stroke	0.2918	1	No	0.2082	Unimpactful
Thyroid	0.9551	0	No	0.5111	Unimpactful

### 4.1 Case 1: National Breast Cancer Awareness Month

One out of eight women in the USA are diagnosed with breast cancer (ACS), and breast cancer is the top cause of cancer death for women 40 to 50 years of age (SEER) and the second leading cause of cancer death for women in the USA (Centers for Disease Control Prevention, 2014). The National Breast Cancer Awareness Event is dedicated to drawing public attention on prevention and early detection, supporting the patients and fundraising for scientific research.

**Fig. 2:**
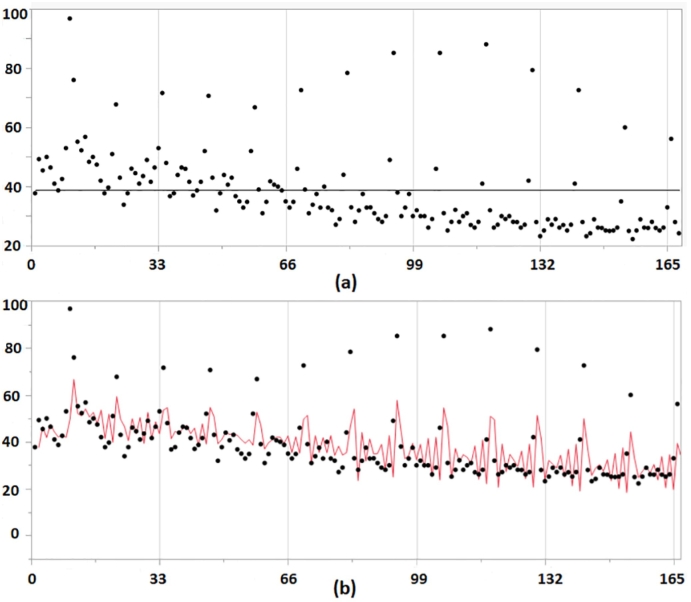
Breast Cancer: (a) shows a Time Series Plot; (b) shows the fitted ARIMA line.

The time series plot as shown in Fig. 2 presented peaks at the event months, October. Three different tests including periodogram, auto-correlation function, and linear model comparison were conducted to check the seasonality. For breast cancer data, two of the three tests indicated that there was no seasonality, therefore we chose ARIMA model instead of SARIMA and obtained the best ARIMA model and transfer function model.

The results were shown in Table 2. Notice that the Adjust *R*^2^ was 0.41 for the ARIMA model and was 0.58 for the transfer function noise model, and the p-value for {*x*_*t*_} parameter “eventmonth” was < 0.0001. Therefore we concluded that the event had a significant effect on the number of search for breast cancer.

**Table 2:** Results for ARIMA and transfer function model(ARIMAX).

	Orders	Adjusted R square	p value of event coefficient
ARIMA	(2,1,3)	0.408	NA
ARIMAX	(2,0,3)	0.583	<0.001

Next, for Wilcoxon rank sum test, the data were split into event month subset and non event month subset. A p-value 0.0000 < 0.05 indicated a rejection to null hypothesis that two groups of observations come from the same population. A larger mean showed that during event months the search frequencies were higher than the rest of the year.

**Fig. 3:**
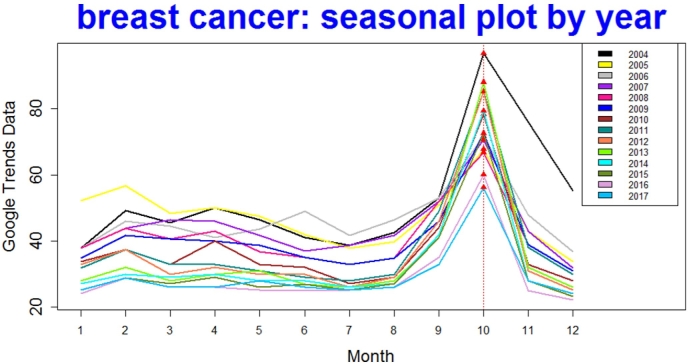
Breast cancer: all 14 peaks fall in October. (For interpretation of the references to color in this figure, the reader is referred to the web version of this article.)

For the Binomial approach, among 14 years of Google Trends data of the query breast cancer, all 14 yearly peaks happened in October (see color Fig. 3). There was evidence to conclude that event-month frequencies were greater than the other months.

In sum, all our results consistently indicated that the National Breast Cancer Awareness event was impactful in increasing search frequency of breast cancer in October.

### 4.2 Case 2: American Stroke Awareness Month

Strokes are one of the leading causes of death and serious long-term disability in the USA (Dariush and et al., 2015). More than 795,000 Americans have a stroke every year and about 130,000 people have been killed by a stroke in the USA each year (Centers for Disease Control and Prevention and NCHS, 2015).

**Fig. 4:**
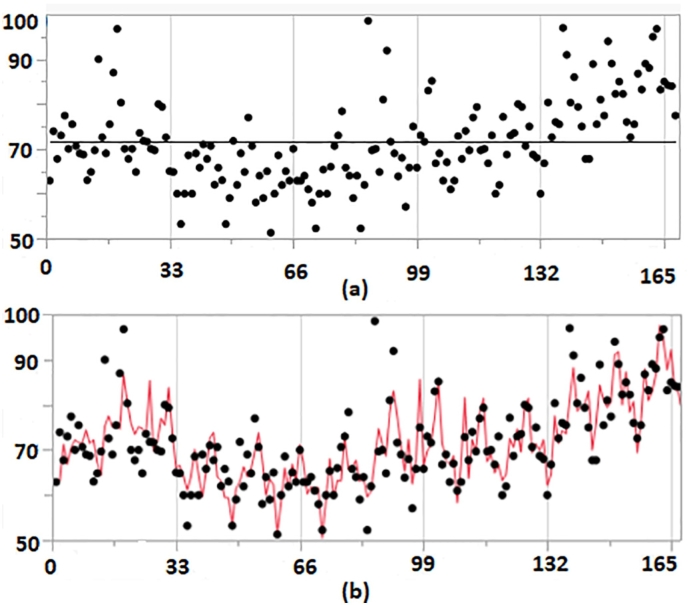
Stroke: (a) shows a Time Series Plot; (b) shows the fitted SARIMA line.

From the GT data of query “strock ”, its time series plot was shown in Fig. 4 (a). Three different tests including peridogram, auto-correlation function, and linear model comparison were used to check the seasonality and all three tests indicated that there was seasonality, meaning SARIMA model should be used. The outputs for SARIMA model and transfer function noise model were presented in Table 3. Notice that the Adjust *R*^2^ was about 0.62 for the transfer function noise model which was no better than the adjust *R*^2^ = 0.68 for SARIMA model, and the p-value for {*x*_*t*_} parameter “eventmonth” was about 0.235 > 0.05. Therefore there was no evidence to conclude that the event had a significant effect on the number of search for Stroke.

**Table 3:** Results for ARIMA and Transfer Function Model(ARIMAX).

	Orders	Adjusted R square	p value of event coefficient
SARIMA	(4,1,2)(2,0,0)	0.677	NA
ARIMAX	(4,1,2)(2,0,0)	0.620	0.2354

One-side Wilcoxon Rank-Sum test had p-value= 0.2918 > 0.05, thus the search frequencies for query “strokes” were not significantly higher in the event month.

**Fig. 5:**
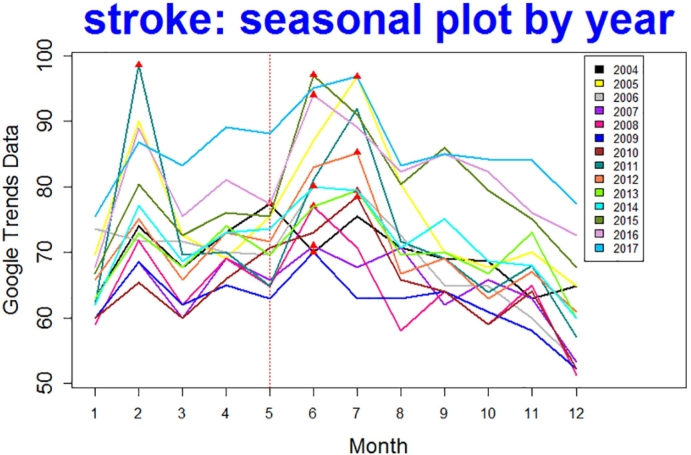
Stroke: one peak falls in May. (For interpretation of the references to color in this figure, the reader is referred to the web version of this article.)

From the years 2004 to 2017, there was only one peak in May (see color Fig. 5) which was less than the threshold 4. In sum, all our results consistently indicated that the there was no evidence that the Stroke Awareness event was impactful in increasing search frequencies of stroke in May.

## 5 Conclusion, limitation and discussion

According to the analysis of all 46 data sets, we found that 10 health awareness events were impactful health awareness events, 28 events were classified as unimpactful health awareness events and the rest were as unclear.

Although in the Google Trend website, only five queries might be compared at once, the authors found a way to compare all the frequencies as follows.

• By searching five queries at a time, the authors obtained an ordered list of the queries by their mean frequencies (see Appendix B).
• Glaucoma was selected as a “Benchmark” query, so any other query was compared to it to generate the relative search frequency data. So we obtained 45 data sets, each of which contained the relative frequencies of Glaucoma and another query.
• Scale the frequencies of Glaucoma so all of the 45 data sets are the same, then combine the resulting frequencies of other 45 queries. So all frequencies are now comparable. Notice that the largest frequency is the combined data is more than 100 because of the rescaling.

The reason of selecting Glaucoma was that it is at a middle-low position. So if it was compared with others, the low frequent ones were still meaningful numbers (i.e. they are showing as “ 0” s, or “ <1 ”), while the high frequent ones were not too large.

The mean frequencies for impactful events, unclear events and unimpactful events were compared pairwisely, and the results were shown in Table 4. Therefore, no class had a significantly higher mean search frequency than another.

**Table 4:** Top: summary statistics for three classes; Bottom: p-values for pairwise t-tests for means.

	Impactful	Unclear	Unimpactful
Mean	115.152	201.313	134.662
SD	142.367	229.026	129.867

			
	Impactful	Unclear	Unimpactful

Impactful	N/A	0.372	0.709
Unclear	0.372	N/A	0.454
Unimpactful	0.709	0.454	N/A

All information and conclusion were entirely from data and statistics perspective. However, statistical significance does not always imply practical significance. For example, the data set has no information about the prevention programming or behaviors among people, therefore it does not distinguish people who barely searched some information and people who learned from the events and started to make changes. The study only analyzed the current months of the events without considering the long term effect, therefore the results only referred to immediate effects.

## Figures and Tables

**Table:** 

Health awareness event/month	Query
*January*	
National Birth Defects Prevention Month	Birth defects
Cervical Health Awareness Month	Cervical
National Glaucoma Awareness Month	Glaucoma
Thyroid Awareness Month	Thyroid

*February*	
American Heart Month	Heart disease
National Children's Dental Health Month	Dental health

*March*	
National Colorectal Cancer Awareness Month	Colon cancer
National Endometriosis Awareness Month	Endometriosis
National Nutrition Month	National nutrition
Multiple Sclerosis Education Month	Sclerosis

*April*	
Alcohol Awareness Month	Alcohol awareness
National Autism Awareness Month	Autism
Irritable Bowel Syndrome Month	Ibs

*May*	
American Stroke Awareness Month	Stroke
Arthritis Awareness Month	Arthritis
National Asthma and Allergy Awareness Month	Asthma allergy
National Celiac Disease Awareness Month	Celiac
Hepatitis Awareness Month	Hepatitis
National High Blood Pressure Education Month	High blood pressure
Lupus Awareness Month	Lupus
Mental Health Month	Mental health
National Osteoporosis Awareness Month	Osteoporosis
Skin Cancer Detection and Prevention Month	Skin cancer

*June*	
National Aphasia Awareness Month	Aphasia
Scoliosis Awareness Month	Scoliosis

*July*	
Eye Injury Prevention Month	Eye injury

*August*	
Amblyopia Awareness Month	Amblyopia
National Immunization Awareness Month	Immunization
Psoriasis Awareness Month	Psoriasis

*September*	
National Alcohol and Drug Addiction Recovery Month	Alcohol drug addiction
National Cholesterol Education Month	Cholesterol
Leukemia and Lymphomn Awareness Month	Leukemia
National Menopause Awareness Month	Menopause
Ovarian Cancer Awareness Month	Ovarian cancer
Prostate Awareness Month	Prostate

*October*	
National Breast Cancer Awareness Month	Breast cancer
National Dental Hygiene Month	Dental hygiene
National Depression and Mental Health Screening Month	Depression
National Down Syndrome Awareness Month	Down syndrome
SIDS Awareness Month	Sids
Spina Bifida Awareness Month	Spina bifida

*November*	
National Alzheimer's Disease Awareness Month	Alzheimer
American Diabetes Month	Diabetes
National Epilepsy Awareness Month	Epilepsy
Lung Cancer Awareness Month	Lung cancer
Pancreatic Cancer Awareness Month	Pancreatic cancer

**Table B1:**
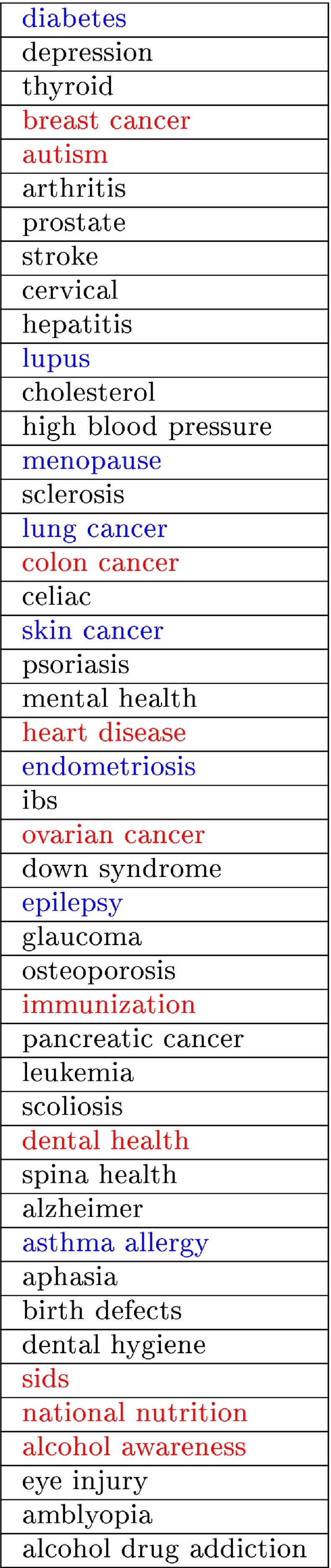
: Impactful; : Unclear; Black: Unimpactful.
